# Analysis of Habituation Learning in Mealworm Pupae (*Tenebrio molitor*)

**DOI:** 10.3389/fpsyg.2021.745866

**Published:** 2021-10-14

**Authors:** Rodolfo Bernal-Gamboa, Jesús García-Salazar, A. Matías Gámez

**Affiliations:** ^1^Facultad de Psicología, Universidad Nacional Autónoma de México, México City, Mexico; ^2^Departamento de Psicología, Universidad de Córdoba, Córdoba, Spain; ^3^Departamento de Psicología, Universidad de Jaén, Jaén, Spain

**Keywords:** dishabituation, habituation, Holometabolous insects, spontaneous recovery, *Tenebrio molitor*

## Abstract

The decline of response as a consequence of repeated stimulation is known as habituation. The goal of the present experiments was extending the knowledge about habituation of abdominal contractions in the pupa of *Tenebrio molitor*. Both experiments consisted of two phases. During Phase 1, all groups were exposed to a continuous stimulus (light in Experiment 1 and vibration in Experiment 2). At the beginning of this phase, pupae showed a high number of abdominal contractions. However, during the last minute of Phase 1, the number of abdominal contractions was lower. In the next phase, the pupae were divided in different groups to test for response recovery. We found an increase in the abdominal contractions when subjects were exposed to a different stimulus, be it within the same or in a distinct sensory modality. In addition, we also reported response recovery when the pupae were re-exposed to the original stimuli after a resting period. Results indicate that the increase in responding cannot be explained by either sensory adaptation or fatigue. The findings are consistent with the perspective that suggests that habituation plays a major role in the survival of the species, even in non-feeding developmental stages.

## Introduction

Habituation can be defined as a gradual decrease in response, caused by the repeated presentation of a stimulus. It can be differentiated from sensory adaptation (i.e., the organs involved in the detection of the stimulus have a decrease in their sensitivity) or motor fatigue (i.e., if the muscles involved are exhausted due to repeated stimulation; see Harris, 1943; Rankin et al., 2009). Because habituation is a widely spread phenomenon in animals, it has been proposed that one of its main functions might be preventing animals from spending unnecessary time and energy on behaviors that are not functional (e.g., Bouton, 2016).

The behavioral characteristics of habituation are well established (Thompson and Spencer, 1966; Rankin et al., 2009): (1) Repeated exposure to a stimulus results in a progressive decrease in the frequency and/or magnitude of a response at an asymptotic level. (2) Habituation dissipates over time. If the stimulus is withheld after the decrease in responding, the response partially recovers (spontaneous recovery). (3) After several repetition cycles of the stimulus and spontaneous recovery, the decrease in response becomes successively faster and/or more pronounced. (4) The magnitude of habituation depends on the interstimulus interval (ISI). A faster but less resistant habituation is produced by shorter ISIs compared to a habituation effect produced by longer ISIs. (5) Habituation is dependent on the intensity of the stimulus. A more rapid and pronounced response decrease is produced by a less intense stimulus. (6) The effect of repeated stimulation can continue cumulatively even after the response has reached its asymptotic level. (7) Within the same stimulus modality, the decrease in response shows some stimulus specificity. (8) The dishabituation effect (recovery of the response to the original stimulus) is produced by the presentation of a different stimulus. (9) Habituation of dishabituation may occur through repeated presentation of the dishabituation stimulus. (10) There are two types of habituation: short-term habituation (the response decrease within a test session that can last from a few seconds to hours) and long-term habituation (a response decrement produced by specific repeating protocols that often require longer ISIs, and it can last from hours to even weeks). Additionally, there is evidence that shows that habituation can be specific to the context where stimulation occurs, which suggests that associative learning might play a role (e.g., Reyes-Jiménez et al., 2020; Dissegna et al., 2021).

The above-mentioned behavioral characteristics summarize the effort of almost a century studying the habituation effect (Harris, 1943; see Thompson, 2009). Currently, they serve as the basis for continuing different lines of research that involve habituation. For example, some authors use information of these characteristics to understand the neural mechanisms underlying habituation (e.g., Byrne et al., 2009; Ardiel and Rankin, 2010; McDiarmid et al., 2019). Other research uses knowledge of behavioral characteristics to contrast the predictions of different theories (e.g., Thompson, 2009; Hall and Rodriguez, 2017; Hall and Rodríguez, 2020). Additionally, several authors have pointed out that knowledge of the behavioral characteristics of habituation can favor the understanding of complex human situations, such as food intake (for a review see Epstein et al., 2009) or sexual behavior and substance abuse (Domjan, 2015). Moreover, using the behavioral characteristics of habituation has proven to be a good starting point for the development of research interested on learning in little-studied animal species (e.g., Prados et al., 2020; Reyes-Jiménez et al., 2020).

In the case of insects, habituation has been demonstrated in ants (Wiel and Weeks, 1996), honeybees (Braun and Bicker, 1992), and fruit flies (Duerr and Quinn, 1982). Nevertheless, the information remains scarce. For example, a large part of those studies does not have a recent follow-up. Additionally, the largest number of investigations on habituation has focused on the order of Diptera (e.g., flies and mosquitoes; see, Duerr and Quinn, 1982; Corfas and Dudai, 1989) and Hymenoptera (e.g., bees, ants, bumblebees, and wasps; see, Barrass, 1961; Simonds and Plowright, 2004; Varnon et al., 2021). However, little is known about habituation in Coleoptera, the order of insects that contains the most species compared to any other in the animal kingdom. Some studies have shown that habituation occurs in different species from the first moments of life (e.g., Rankin and Carew, 1987, 1988; Chiandetti et al., 2018). Furthermore, habituation has been reported even in embryonic stages of domestic chicks (Turatto et al., 2019). Nevertheless, in insects, most studies have neglected research in the pupal stage, which is often considered an inactive transition point (e. g., Hawes, 2019). Thus, highlighting the habituation effect at this stage (dedicated almost exclusively to the transformation of a larva into an adult) would provide an additional insight of the relevance of this learning process for the survival of a species in any of the developmental stages of its life. For those two reasons, we evaluated habituation in the pupal stage of one insect belonging to the order of the coleopterans, the beetle *Tenebrio molitor*. Specifically, two experiments were designed to assess some behavioral characteristics of the habituation response of abdominal contraction in the pupae of the *Tenebrio molitor,* commonly known as the mealworm.

The mealworm beetle (*Tenebrio molitor*) presents a complete metamorphosis (holometabolo) and goes through four different developmental stages: egg, larva, pupa, and finally adult. It is susceptible to being attacked by various predators during the pupal stage. However, it has various strategies to defend itself. One of them is the circular rotation of the abdominal segments for its defense (Ichikawa and Kurauchi, 2009). Additionally, it has also been reported that abdominal rotations may have a function to escape light stimuli that produce heat (Askew and Kurtz, 1974).

In the first experiment that studied habituation of the abdominal contraction response, the pupae were exposed to two types of stimulation: electrical (i.e., electric shocks administered to the sides of the head) and tactile (i.e., rubbing the body of the pupal head with a camel hairbrush to the thorax). After the pupae showed habituation to the electrical stimulation, they were alternately exposed to electrical stimulation and tactile stimulation. The results indicated higher levels of abdominal contractions when the pupae were exposed to the new stimulation (tactile) compared to the original electrical stimulation (Hollis, 1963). Hollis’s results were replicated and extended by Askew and Kurtz (1974) to a situation that showed a slower habituation when a more intense electrical stimulation (50μa) was used compared to less intense electrical stimulation (4μa; Experiment 3). Additionally, those authors observed the spontaneous recovery effect (Experiment 4).

Somberg et al. (1973) evaluated the gradual decrease in abdominal contractions using light stimulation. The habituation phase in their experiment consisted of exposing the pupae for 13min to continuous light. Then, half of the pupae received 13min of flashing light, while the other half remained in darkness for 60min, and subsequently received another 13min of continuous light. The authors reported that both groups showed an increase in the number of abdominal contractions, indicating both stimulus specificity and spontaneous recovery of the habituated response.

While the aforementioned studies presented findings on the habituation effect in the *Tenebrio molitor* pupa, it is important to highlight that the evidence is still scarce. Moreover, as far as the authors know there has not been subsequent reports studying this effect in the pupae since the earlier 70s of the last century. Thus, to stablish a valid procedure to further evaluating the mechanisms (neurobiological and psychological) involved in the habituation of this species’ pupa, a replication of the basic findings should be conducted. Therefore, the proposal of this research was to follow up and extend the study of habituation in the pupa of *Tenebrio molitor*. We based our procedure on Somberg et al. (1973). However, since those authors did not describe in detail the experimental apparatus used, we built an experimental chamber to place the insects safely, and in which they received the presentation of the stimuli.

## Experiment 1

In Experiment 1 (see upper part of Table 1), four groups of pupae were exposed to a continuous light for 13min in Phase 1. Then, in Phase 2, Groups L-FL, L-V, and L-L were immediately exposed to a period of 13min of flashing light, vibration, or a continuous light stimulation, respectively, whereas pupae in Group L/L were exposed to 13min of continuous light after 60min without stimulation (they were left in the experimental chamber, with the lights off). A decline of the number of abdominal contractions was expected for all groups in Phase 1. If the response decline was due to habituation, we should observe in Phase 2 an increment in the response level in all groups, except for Group L-L.

**Table 1:** Experimental designs.

Experiment	Group	Phase 1	Resting period	Phase 2
*1*	L/L	L	60min in the dark	L
	L-FL	L	0min	FL
	L-V	L	0min	V
	L-L	L	0min	L
*2*	V/V	V	60min without vibration	V
	V-L	V	0min	L
	V-V	V	0min	V

*Stimuli: L=continuous light, FL=flashing light, and V=vibration. See text for details.*

### Method

#### Participants

Fifty-two pupae of *Tenebrio molitor* were used (13 per group). They were approximately 5hours old (as pupae) at the beginning of the experiment. Larvae were housed individually in plastic compartments (3.3×3.3×2.5cm; height×width×depth) and were fed with wheat bran, oats, and carrot fragments. They were kept inside a room maintained on a 12–12h light-dark cycle (07:00 onset and 19:00 offset of lights). The temperature of the room ranged between 20 and 25°C, while the humidity value was 45–60%. The present experimental protocol was conducted under strict agreement with the guidelines established by the Ethical Committee of the Faculty of Psychology of the National University of Mexico and the Ethical Code of the Mexican Society of Psychology.

#### Apparatus and Stimuli

A 4mm MDF wood chamber with white melamine of 4×7.2×5.5cm (W x L x H) was built. The front wall was removed for the recordings of the contractions, and the ceiling was removable. The base was made of cardboard and 1cm high. A 1.1×2×2cm (W × L × H) foam platform was glued into it, on which the pupae were attached with transparent adhesive tape. For the vibratory stimulation, one mini vibratory motor (3Vdc, 90ma, 12,000±2,500 RPM, 10mm diameter, and 3mm wide) was connected to a 1 KΩ potentiometer under the base to be able to regulate the intensity of the vibrations. The frequency of the vibrations was 200 times per minute.

**Figure 1:**
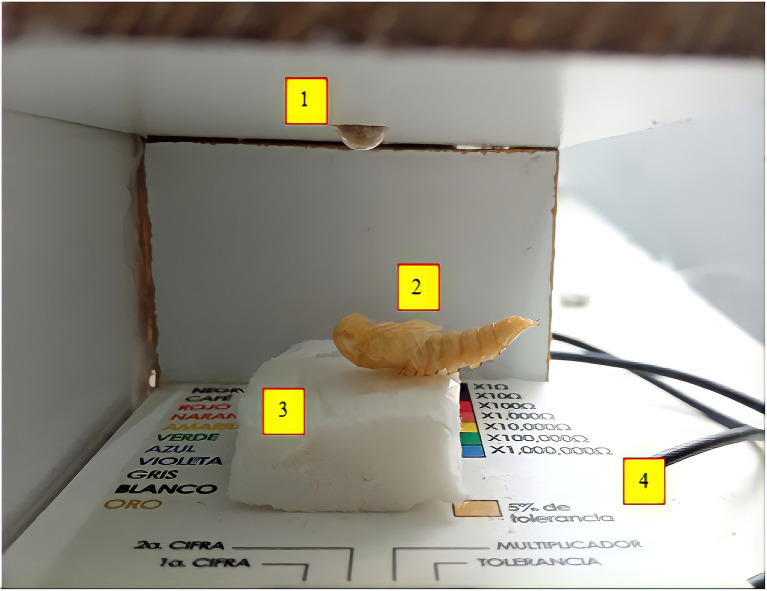
Experimental chamber. 1=LED lights, 2=pupae, 3=foam platform, and 4=mini vibrating motor.

For the continuous light stimulation, two ultra-bright white LEDs were used (head diameter 5mm; pin length, 8.6mm; 2.8–3.6V; and electric current 20ma), each with a resistance of 330Ω. For the flashing lights, an electrical circuit was built in a breadboard capable of controlling the two ultra-bright white LEDs (head diameter 5mm; pin length, 8.6mm; 2.8–3.6V; and electric current 20ma), which consisted of 1N555 integrated circuit, 1,330, 560, 1 KΩ resistor, 1 2N2222A transistor, 1100 mF capacitor, and a 15 KΩ potentiometer. The light was flashing 636 times per minute, and this flashing stimulation was constant throughout the 13min. All the LEDs were at a distance of 1.5cm from the pupae. There was an individual switch for the LEDs and the mini vibrating motor. The power supply was 9Vdc 300ma (see Figure 1).

All sessions were recorded with a compact digital camera (Sony DSC-W800) mounted on a 22cm high metal tripod (Solidex TR-1). One researcher observed the video recordings and counted the contractions that started within a minute and ended in the minute after, given that abdominal contractions decreased mostly from minute to minute. The sessions were conducted in a room lit by a 23W white fluorescent light bulb.

#### Procedure

When the activity of the larvae diminished, becoming almost zero, they were separated from the colony and placed in a container with wheat bran. After their last larval ecdysis, a period of 4h minimum and 5h maximum was allowed to elapse, in which the pupae were kept in a box to avoid light. The sessions began once this period had elapsed, in which the dorsal part of the thorax of the pupae was fixed with adhesive tape to the foam platform.

The experiment consisted of two phases, which are described below:

##### Phase 1

All the pupae were placed in the experimental apparatus, in which they received 13min of uninterrupted light stimulation (during this phase, two white LED lights were kept constantly on).

##### Phase 2

For Groups L-FL, L-V, and L-L, this phase was conducted immediately after the last minute of the previous phase, while the pupae in Group L/L were exposed to this phase after 60min without stimulation (the lights were turned off and they were left in the experimental chamber). Both Group L/L and Group L-L were exposed in this second phase to the same stimulation as in the previous phase (i.e., 13min of continuous light). For Group L-FL, pupae received 13min of light stimulation, but unlike Phase 1, in this second experimental phase, the light was not fixed but intermittent (i.e., the two lights were flashing). Finally, Phase 2 for Group L-V consisted of receiving only 13min of vibratory stimulation (the lights were no longer lit for these pupae).

Thus, Group L/L and Group L-FL replicate the experimental groups used by Somberg et al. (1973), while groups L-V and L-L were added for the present research.

#### Dependent Variable and Statistical Analysis

Abdominal contractions per minute were analyzed using a mixed ANOVA. The rejection criterion was set at *p*<0.05, and effect sizes were reported using partial eta-squared ($\eta_{p}{}^{2}$). For measures of effect size, 95% confidence intervals (CIs) were computed using the method reported by Nelson (2016). For the multiple post-hoc comparisons, the Bonferroni correction was used when the assumption of homogeneity of variances was fulfilled, otherwise the Games-Howell correction was used. The Greenhouse-Geisser correction was used when the assumption of sphericity was not fulfilled.

### Results and Discussion

**Figure 2:**
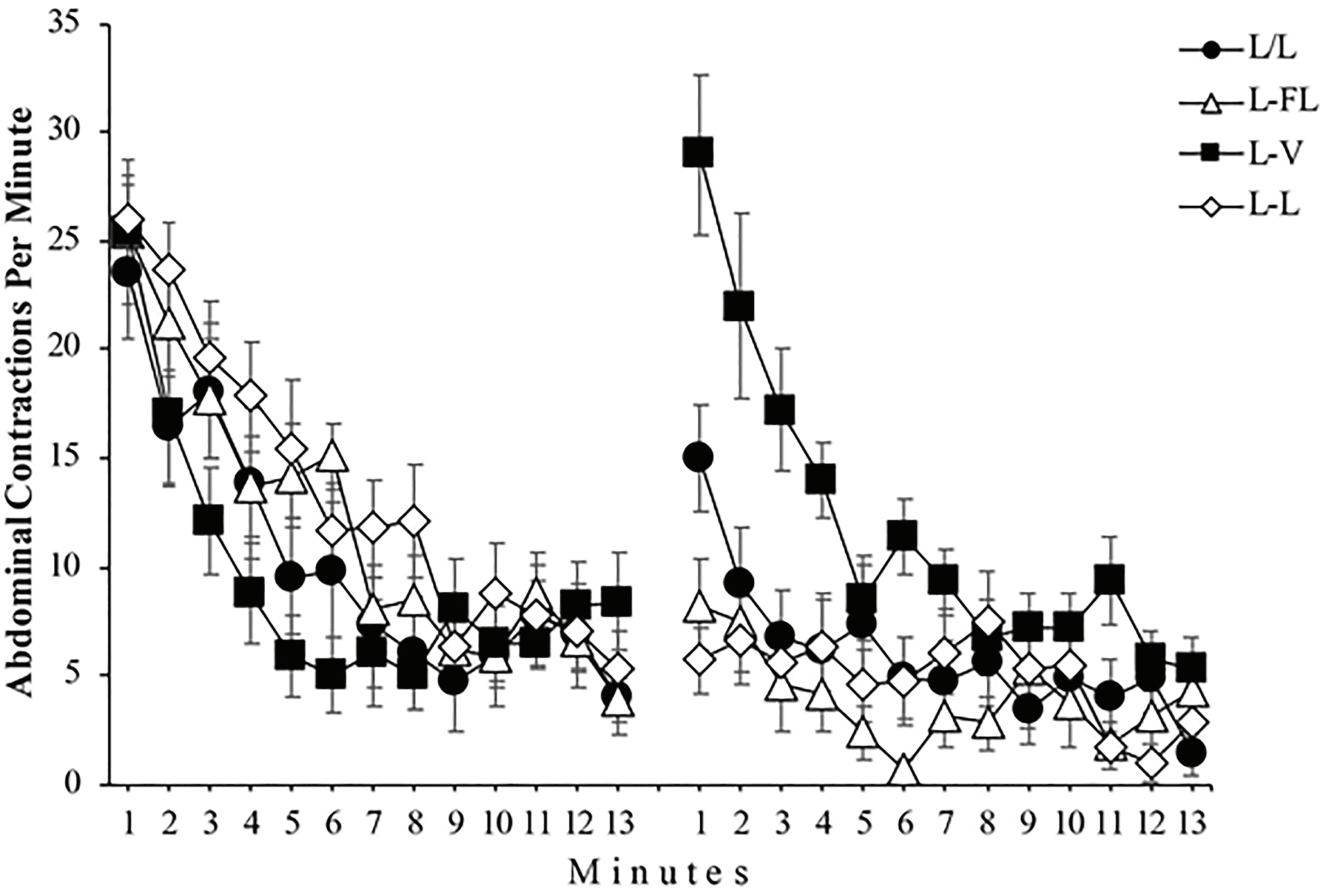
Mean abdominal contractions during the 13 minutes of Phase 1 (left panel) and the 13 minutes of Phase 2 (right panel) of Experiment 1. Error bars denote standard errors of the mean.

Figure 2 depicts the mean abdominal contractions per minute throughout Phase 1 and 2 for all groups. A mixed 4 (Group) x 2 (Phase) x 13 (Minute) ANOVA found a significant effect of Phase, *F*(1, 48)=41.67, *p<0*.001, $\eta_{p}{}^{2}$=0.46, 95% CI [0.25,0.61], and Minute, *F*(5.73, 275.21)=44.41, *p<0*.001, $\eta_{p}{}^{2}$=0.48, 95% CI [0.39,0.54]. The Group x Phase interaction, *F*(3, 48)=12.26, *p<0*.001, $\eta_{p}{}^{2}$=0.43, 95% CI [0.19,0.56], the Group x Minute interaction, *F*(36, 576)=1.60, *p=0*.016, $\eta_{p}{}^{2}$=0.09, 95% CI [0.00,0.09], and the Phase x Minute interaction, *F*(7.75, 372.08)=6.10, *p<0*.001, $\eta_{p}{}^{2}$=0.11, 95% CI [0.04,0.16] were also significant. More importantly, the triple Group × Phase × Minute interaction did reach significance, *F*(23.25, 372.08)=2.90, *p<0*.001, $\eta_{p}{}^{2}$=0.15, 95% CI [0.04,0.17].

For a detailed analysis of this triple interaction, we begin with the analysis of Phase 1. A similar response pattern in all four groups can be seen in the left panel of Figure 2: The number of abdominal contractions decreases as minutes go by. A mixed 4 (Group) x 13 (Minute) ANOVA conducted with the Phase 1 data found a significant effect of Minute, *F*(6.29, 302.34)=43.18, *p<0*.001, $\eta_{p}{}^{2}$=0.47, 95% CI [0.39,0.53], while neither the main effect of Group, *F*(3, 48)=1.32, *p=0*.278, $\eta_{p}{}^{2}$=0.07, nor the Group x Minute interaction, *F*(18.89, 302.34)=1.55, *p=0*.068, $\eta_{p}{}^{2}$=0.08, reached significance.

**Figure 3:**
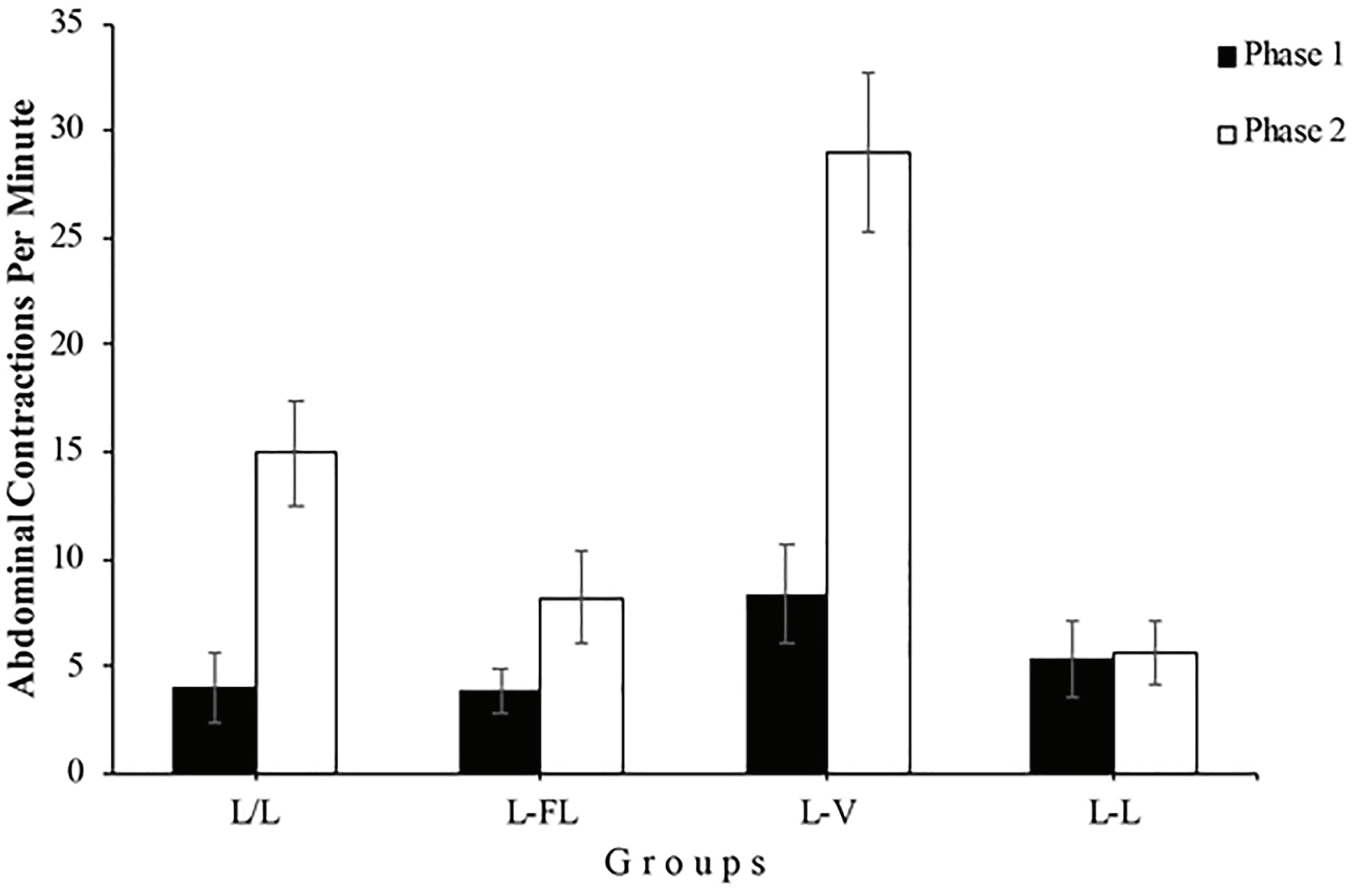
Mean abdominal contractions during the last minute of Phase 1 (black bars) and the first minute of Phase 2 (white bars) of Experiment 1. Error bars denote standard errors of the mean.

The right panel of Figure 2 shows that the number of abdominal contractions in Phase 2 is different depending on the group, especially during the first minutes of this phase. A mixed 4 (Group) x 13 (Minute) ANOVA conducted with the Phase 2 data yielded a significant main effect of Group, *F*(3, 48)=15.57, *p<0*.001, $\eta_{p}{}^{2}$=0.49, 95% CI [0.26,0.61] and Minute, *F*(7.39, 354.98)=12.72, *p<0*.001, $\eta_{p}{}^{2}$=0.21, 95% CI [0.13,0.27]. Moreover, the Group x Minute interaction was significant as well, *F*(22.18, 354.98)=2.77, *p<0*.001, $\eta_{p}{}^{2}$=0.15, 95% CI [0.04,0.16]. In a detailed analysis of this interaction, two one-way ANOVAs found that the simple effect of Group was significant in minute 1, *F*(3, 48)=16.01, *p<0*.001, $\eta_{p}{}^{2}$=0.50, 95% CI [0.26,0.62] but not in minute 13, *F*(3, 48)=1.95, *p=0*.134, respectively, which indicates that the number of abdominal contractions during the first minute of Phase 2 was different between groups, whereas this number was similar among the groups in the 13th minute. Specifically, post-hoc analyses showed significant differences in minute 1 between L/L and L-V groups, *p*=0.022, L/L and L-L groups, *p*=0.019, L-FL and L-V groups, *p*=0.001, and L-V and L-L groups, *p*=0.000 (see Figure 3).

On the analysis of the results for each group, using the data from Group L/L, we found a significant increase in the number of abdominal contractions in the first minute of Phase 2 compared to the last minute of Phase 1, *F*(1, 12)=15.92, *p=0*.002, $\eta_{p}{}^{2}$=0.57, 95% CI [0.13,0.75], showing the spontaneous recovery of the abdominal contractions response as a consequence of the resting period between the two phases. The analysis also found a significant difference in the number of abdominal contractions between phases in Group L-FL, *F*(1, 12)=5.82, *p=0*.033, $\eta_{p}{}^{2}$=0.33, 95% CI [0.00,0.60]. This indicates that presenting an intermittent light instead of a continuous light during Phase 2 also produced an increment in the number of abdominal contractions, regardless of the fact that Group L-FL was immediately exposed to the flashing light with no resting period. As can be seen in Figure 3, Group L-V shows a higher increase in the number of abdominal contractions between the end of Phase 1 and the beginning of Phase 2, *F*(1, 12)=21.84, *p<0*.001, $\eta_{p}{}^{2}$=0.64, 95% CI [0.21,0.79], as a consequence of changing from continuous light to vibration. Finally, as expected, there were no significant differences in the number of abdominal contractions between both phases in Group L-L, *F*<1. At this point, group L-FL and group L-V results could be an evidence of stimulus specificity of habituation, although vibration seems to be perceived much differently from continuous light than is flashing light.

Lastly, we analyzed the level of habituation showed by each group during Phase 2 (see right panel of Figure 2). The detailed analysis of the Group x Minute interaction conducted with the data from Phase 2 yielded a significant simple effect of Minute in Group L/L, *F*(3.93, 47.23)=3.90, *p=0*.008, $\eta_{p}{}^{2}$=0.24, 95% CI [0.02,0.38], indicating rehabituation of the abdominal contraction response to the continuous light in this second phase. The simple effect of Minute was also significant in Group L-V, *F*(4.58, 54.98)=10.46, *p<0*.001, $\eta_{p}{}^{2}$=0.47, 95% CI [0.23,0.57], which indicates that the pupae habituated to the vibrations. The simple effect of Minute did not reach significance in Group L-FL, *F*(4.66, 56.01)=1.85, *p=0*.121, $\eta_{p}{}^{2}$=0.13, showing no abdominal contractions response habituation to the flashing light. Nevertheless, a significant difference was found by comparing the number of abdominal contractions between the first minute and the last minute of this phase, *F*(1, 12)=5.05, *p=0*.044, $\eta_{p}{}^{2}$=0.30, 95% CI [0.00,0.58]. Finally, the simple effect of Minute was not significant in the group L-L, *F*(5.26, 63.17)=1.34, *p=0*.254, $\eta_{p}{}^{2}$=0.10, demonstrating that the habituation to the continuous light reached an asymptotic level at the beginning of the second phase.

## Experiment 2

In Experiment 1, we demonstrated that after habituation to a light stimulation was established, a response recovery could be reported when pupae were exposed to a distinct light stimulation, to a new vibration stimulus, or to the original light stimulus but after a resting period. Given that vibrations are also part of the surroundings of the *Tenebrio molitor* in the pupa stage and that we found habituation to that stimulation in the previous experiment, the aim of the present experimental design was to further evaluate whether the recovery of the habituation found in Experiment 1 could be extended to a vibratory stimulation.

In Experiment 2 (see lower part of Table 1), three groups of pupae were exposed to vibration for 13min in Phase 1. Next, during the 13min of Phase 2, pupae in Groups V/V and V-V were re-exposed to vibratory stimulation, while pupae in Group V-L received a light stimulation. Groups V-L and V-V received Phase 2 immediately after Phase 1. For pupae in Group V/V, Phase 2 was conducted after 60min without stimulation (they were left in the experimental chamber without vibration). We expected a similar habituation in all three groups in Phase 1. We also expected response recovery during Phase 2 for Groups V/V and V-L only.

### Method

#### Participants

Thirty-nine pupae of *Tenebrio molitor* were used (13 per group). They were approximately 5hours old at the beginning of the experiment. The rest of characteristics is the same as those in the previous experiment.

#### Apparatus and Stimuli

We conducted the present experiment in the same conditions as in Experiment 1.

#### Procedure

Except as noted, we used the same procedure as in Experiment 1.

##### Phase 1

The pupae were placed in the experimental apparatus, in which they received 13min of uninterrupted vibratory stimulation.

##### Phase 2

For Groups V-L and V-V, this phase was conducted immediately after the last minute of the previous phase, while this phase was conducted for the pupae in Group V/V after 60min without stimulation had elapsed (they were left in the experimental apparatus, without vibratory stimulation). Both Groups V/V and V-V were exposed in this second phase to the same stimulation as in the previous phase (i.e., 13min of vibration). For Group V-L, pupae received 13min of continuous light stimulation.

### Results and Discussion

**Figure 4:**
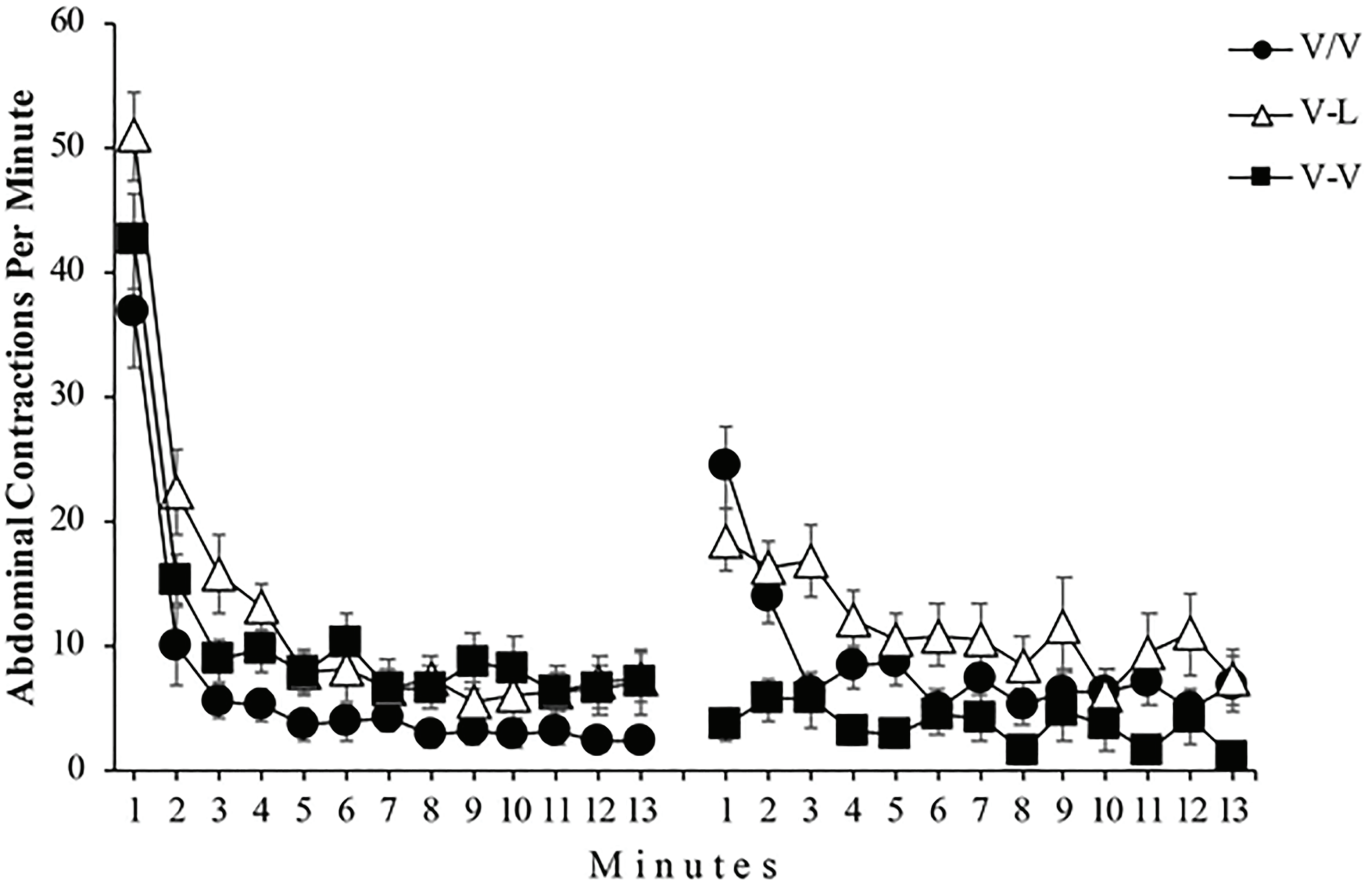
Mean abdominal contractions during the 13 minutes of Phase 1 (left panel) and the 13 minutes of Phase 2 (right panel) of Experiment 2. Error bars denote standard errors of the mean.

Figure 4 shows the mean abdominal contractions per minute throughout both phases of the experiment for all subjects. A mixed 3 (Group) x 2 (Phase) x 13 (Minute) ANOVA found a significant main effect of Group, *F*(2, 36)=6.42, *p=0*.004, $\eta_{p}{}^{2}$=0.26, 95% CI [0.03,0.44], Phase, *F*(1, 36)=7.10, *p=0*.011, $\eta_{p}{}^{2}$=0.16, 95% CI [0.01,0.37], and Minute, *F*(6.03, 217.30)=77.07, *p<0*.001, $\eta_{p}{}^{2}$=0.68, 95% CI [0.61,0.72]. In addition, the Group x Phase, *F*(2, 36)=10.88, *p<0*.001, $\eta_{p}{}^{2}$=0.37, 95% CI [0.11,0.54], the Group x Minute, *F*(12.07, 217.30)=2.27, *p=0*.010, $\eta_{p}{}^{2}$=0.11, 95% CI [0.01,0.15], and the Phase x Minute, *F*(6.78, 244.40)=28.54, *p<0*.001, $\eta_{p}{}^{2}$=0.44, 95% CI [0.34,0.50] interactions reached significance. Moreover, the triple Group x Phase x Minute interaction was also significant, *F*(13.57, 244.40)=1.86, *p=0*.032, $\eta_{p}{}^{2}$=0.09, 95% CI [0.00,0.12]. For a subsequent detailed analysis of the triple interaction Group x Phase x Minute, we first analyzed the data of Phase 1. The left panel of Figure 4 depicts that all three groups performed similarly throughout this phase by showing that the number of abdominal contractions drops dramatically in the first few minutes of exposure to vibrations. A mixed 3 (Group) x 13 (Minute) ANOVA conducted with the Phase 1 data found significant main effects of Group and Minute, *F*(2, 36)=8.80, *p=0*.001, $\eta_{p}{}^{2}$=0.33, 95% CI [0.08,0.50] and *F*(4.86, 175.20)=84.06, *p<0*.001, $\eta_{p}{}^{2}$=0.70, 95% CI [0.62,0.74], respectively. However, the Group x Minute interaction did not reach significance, *F*(9.73, 175.20)=1.31, *p=0*.226, $\eta_{p}{}^{2}$=0.07. Moreover, a one-way ANOVA found no significant differences between the three groups in the first and in the last minute of Phase 1, *F*(2, 36)=3.16, *p*=0.057 and *F*(2, 36)=1.99, *p*=151, respectively. Since there appears to be no difference between groups at the beginning and end of Phase 1, this result shows that the level of habituation was similar for all groups.

The right panel of Figure 4 shows that the performance of the groups was different during Phase 2, primarily on the first minutes of this phase. A mixed 3 (Group) x 13 (Minutes) ANOVA conducted with the Phase 2 data found a significant main effect of Group, *F*(2, 36)=7.40, *p=0*.002, $\eta_{p}{}^{2}$=0.29, 95% CI [0.05,0.47]. The main effect of Minute was also significant, *F*(8.41, 302.82)=8.58, *p<0*.001, $\eta_{p}{}^{2}$=0.21, 95% CI [0.10,0.25]. Most importantly for the present experiment, the Group x Minute interaction also reached significance, *F*(16.82, 302.82)=3.14, *p<0*.001, $\eta_{p}{}^{2}$=0.15, 95% CI [0.04,0.18]. Follow-up analysis of this interaction found that the simple effect of Group was significant both in minute 1, *F*(2, 36)=18.12, *p<0*.001, $\eta_{p}{}^{2}$=0.50, 95% CI [0.24,0.64], and in minute 13, *F*(2, 36)=3.63, *p=0*.037, $\eta_{p}{}^{2}$=0.17, 95% CI [0.00,0.35], showing differences between groups at the beginning and at the end of Phase 2. Post-hoc analyses showed significant differences in minute 1 between groups V/V and V-V, *p*=0.000 and between groups V-L and V-V, *p*=0.000; moreover, in minute 13, post-hoc analyses showed significant differences between groups V-L and V-V, *p*=0.034.

Regarding the level of habituation response produced in each group throughout Phase 2, the simple effect of Minute was significant in Group V/V, *F*(4.27, 51.30)=11.56, *p<0*.001, $\eta_{p}{}^{2}$=0.49, 95% CI [0.25,0.60] indicating rehabituation to the vibration, and in Group V-L, *F*(4.94, 59.29)=3.96, *p<0*.001, $\eta_{p}{}^{2}$=0.25, 95% CI [0.03,0.37], demonstrating that subjects were habituated to the light. Nevertheless, the simple effect of Minute was not significant in Group V-V, *F<1*, showing that the number of abdominal contractions did not change along Phase 2 for this group.

**Figure 5:**
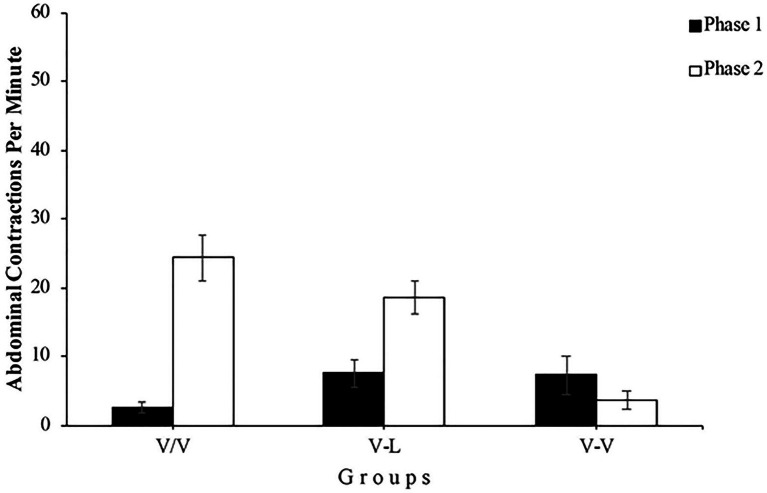
Mean abdominal contractions during the last minute of Phase 1 (black bars) and the first minute of Phase 2 (white bars) of Experiment 2. Error bars denote standard errors of the mean.

During the results analysis for each group (see Figure 5), using the data from Group V/V, we found that the increase of abdominal contractions in the first minute of Phase 2 compared to the last minute of Phase 1 was significant, *F*(1, 12)=33.84, *p<0*.001, $\eta_{p}{}^{2}$=0.74, 95% CI [0.35,0.85], demonstrating the spontaneous recovery of the habituated response. The analysis also reported a significant difference in the number of abdominal contractions between phases in the Group V-L, *F*(1, 12)=16.55, *p=0*.002, $\eta_{p}{}^{2}$=0.58, 95% CI [0.14,0.75]. As in the previous experiment, this result shows that although subjects of this group underwent Phase 2 immediately after Phase 1, changing the type of stimulation between phases leads to a boost in the response of abdominal contractions, showing once again the stimulus specificity of habituation. Finally, Group V-V seems to show a similar number of abdominal contractions in both phases, which should not be surprising since subjects were exposed to the same stimulation. This appreciation was confirmed by the statistical analysis, *F*(1, 12)=1.69, *p=0*.217.

## General Discussion

The present study aimed to contribute to the literature on habituation in insects by replicating and extending the findings of Somberg et al. (1973), showing habituation of abdominal contractions produced by exposure to light and vibratory stimulation in the pupa of *Tenebrio molitor.* Moreover, since we found a robust effect of habituation to distinct kinds of stimuli, even in a developmental stage in which the behavioral capacities are restrained (pupae do not feed, crawl, nor mate), our present data are consistent with the perspective that suggests that habituation plays a key role in the survival of the species (e.g., Álvarez et al., 2017).

As mentioned above, the present work replicates the results of Somberg et al. (1973). However, it is important to note that, since those authors did not describe neither the apparatus nor the intensity of the light stimuli used, the methodological details reported here, such as the characteristics of the experimental chambers and the stimuli used, may favor the systematic study of habituation effect in the pupal developmental stage of *Tenebrio molitor*.

Our data also contribute to the systematic study of habituation in insects by incorporating controls that make it possible to rule out that factors other than habituation are responsible for the decrease in response (Rankin et al., 2009). For example, the decrease in abdominal contractions of the pupae can hardly be explained as sensory adaptation because the pupae in Group L-V (Experiment 1) showed an increase in the number of abdominal contractions in phase 2, when exposed to vibratory stimulation (see Group V-L of Experiment 2 for similar results when exposure to the stimulus was the opposite). Similarly, the possibility of explaining the decrease in abdominal contractions due to sensory fatigue and/or motor fatigue is ruled out when observing that the pupae show an increment of responding to other stimuli; one within the same sensory modality (Group L-FL, Experiment 1) and the other in a different sensory modality (Group L-V, Experiment 1; Group V-L, Experiment 2).

Given the relevance of the behavioral characteristics of habituation described above, it worth to mention the characteristics obtained so far. We reported data consistent with Characteristic 1 (e.g., all groups in Phase 1 and Group L-V in Phase 2 in Experiment 1), Characteristic 2 (both Groups L/L and V/V in both experiments), and Characteristic 5 (e.g., comparing the decrease observed between all groups during Phase 1 in Experiment 1 and the groups during Phase 1 in Experiment 2). The results of our Group L-L (Experiment 1) and Group V-V (Experiment 2) can be used to exemplify Characteristic 6, because although it can be considered that the level of abdominal contractions reached an asymptote, an even greater decrease was observed in the last trials of Phase 2. Characteristic 7 can be observed in our Group L-FL (Experiment 1) since when pupae were presented with a different stimulus within the same modality (from constant light to flashing light) in Phase 2, abdominal contractions increased. Finally, the results obtained during Phase 2 for Group L-V (Experiment 1) and Group V-L (Experiment 2) are consistent with the so-called cessation effect (Characteristic 8). Therefore, this preliminary study on habituation in *Tenebrio molitor* pupa allowed us to report six of the 10 behavioral characteristics of the habituation effect (see Table 2). Future research might use the method developed here to continue evaluating the rest of the behavioral characteristics (Characteristics 3, 4, 9, and 10).

**Table 2:** Behavioral characteristics of the habituation effect.

Behavioral characteristics of the Habituation effect	Reported in the current study using the pupa of *Tenebrio molitor*
Repeated exposure to a stimulus results in a progressive decrease in the frequency and/or magnitude of a response at an asymptotic level.	Yes
Habituation dissipates over time. If the stimulus is withheld after the decrease in responding, the response partially recovers (spontaneous recovery).	Yes
After several repetition cycles of the stimulus and spontaneous recovery, the decrease in response becomes successively faster and/or more pronounced.	No
The magnitude of habituation depends on the interstimulus interval (ISI). A faster but less resistant habituation is produced by shorter ISIs compared to a habituation effect produced by longer ISIs.	No
Habituation is dependent on the intensity of the stimulus. A more rapid and pronounced response decrease is produced by a less intense stimulus.	Yes
The effect of repeated stimulation can continue cumulatively even after the response has reached its asymptotic level.	Yes
Within the same stimulus modality, the decrease in response shows some stimulus specificity.	Yes
The dishabituation effect (recovery of the response to the original stimulus) is produced by the presentation of a different stimulus.	Yes
Habituation of dishabituation may occur through repeated presentation of the dishabituation stimulus.	No
There are two types of habituation: short-term habituation and long-term habituation.	No

The present experiments may stimulate subsequent studies using the procedure described here to evaluate the psychological (by comparing different theories) and/or neurobiological mechanisms that underlie habituation in coleopterans. Additionally, given that the methodology used in the present study shows that a relatively stable response level to three different stimuli (continuous light, flashing light, and vibrations) can be observed in the pupae, future research may focus on the development of an experimental method that allows the study of Pavlovian learning with these insects in this developmental stage.

## Data Availability Statement

The raw data supporting the conclusions of this article will be made available by the authors, without undue reservation.

## Ethics Statement

The study involving animals was conducted under strict agreement with the guidelines established by the Ethical Committee of the Faculty of Psychology of the National University of Mexico and the Ethical Code of the Mexican Society of Psychology.

## Author Contributions

RB-G: conceptualization, methodology, investigation, writing – original draft, and writing – review and editing. JS: building of experimental chamber and investigation. AG: conceptualization, methodology, formal analysis, writing – original draft, and writing – review and editing. All authors contributed to the article and approved the submitted version.

## Funding

This research was funded by the UNAM/DGAPA through grant project PAPIIT IN306020. Participation of AG was funded by the Junta de Andalucía, Spain, Research grant HUM642.

## Conflict of Interest

The authors declare that the research was conducted in the absence of any commercial or financial relationships that could be construed as a potential conflict of interest.

## Publisher’s Note

All claims expressed in this article are solely those of the authors and do not necessarily represent those of their affiliated organizations, or those of the publisher, the editors and the reviewers. Any product that may be evaluated in this article, or claim that may be made by its manufacturer, is not guaranteed or endorsed by the publisher.
